# Free Rest for Hospital Nurses

**Published:** 1888-05-26

**Authors:** K. Philippa Hicks

**Affiliations:** Lady Supt. of the Hospital for Sick Children, Great Ormond Street, Bloomsbury, W.C.


### FREE REST FOR HOSPITAL NURSES.

We have very great pleasure in commending the following scheme. It is based upon practical knowledge of what is required, and should be as popular with the public for its admirable simplicity as it is likely to prove welcome to the nurses from its kindly considerateness:—

Will you allow me, through your paper, to make more widely known a means of Free Rest for Hospital Nurses. Now that the holiday time is coming on many nurses whose homes are in Scotland or Ireland are unable to afford the long journey, or there are some who have no homes, others who for family reasons are unable to go to their friends. It is to these sisters and nurses I offer this means of rest, partly to prevent their hard-earned savings being spent in the weekly payment of a Convalescent Home, but chiefly that they may benefit by a complete country rest, unfettered by regulations which are to a certain extent necessary in all Homes of Rest. Further particulars may be obtained by letter; or by application to me between the hours of two and four p.m. any afternoon.—I am, Sir, faithfullly yours, K. Philippa Hicks.

Lady Supt. of the Hospital for Sick Children, Great Ormond Street, Bloomsbury, W.C.

